# Intermolecular Hydrogen Bonding in Associated Fluids: The Case of Isopentyl Alcohol Dissolved in Carbon Tetrachloride

**DOI:** 10.3390/molecules28176285

**Published:** 2023-08-28

**Authors:** Stefanos Tsigoias, Constantine Kouderis, Agni Mylona-Kosmas, Angelos G. Kalampounias

**Affiliations:** 1 Department of Chemistry, University of Ioannina, GR-45110 Ioannina, Greece; 2 Institute of Materials Science and Computing, University Research Center of Ioannina (URCI), GR-45110 Ioannina, Greece

**Keywords:** hydrogen bonding, isopentyl alcohol, FTIR, DFT, associated fluids

## Abstract

Fourier-transform infrared (FTIR) spectra of isopentyl-alcohol dissolved in carbon tetrachloride (CCl_4_) were recorded as a function of concentration and temperature. Dilute isopentyl alcohol/CCl_4_ solutions were prepared in alcohol at concentrations of 1, 0.5, 0.3, 0.2, 0.1, 0.05, 0.02, 0.01, 0.005, 0.001 and 0.0005 M. Infrared absorption measurements were taken within a temperature range of 17–67 °C below the boiling point of the solutions. Decomposition of the spectral features corresponding to associated and unassociated species was performed to quantitatively follow the effect of temperature and concentration on intermolecular hydrogen bonding (HB) in isopentyl alcohol. The spectral feature in the 3600–3650 cm^−1^ frequency range attributed to the free OH stretching band was studied in detail to determine changes based on concentration and temperature variations. Computational methodologies were applied to evaluate the energetics and vibrational properties of the species involved in the structure in the gaseous state where no interactions are present. The results are discussed in view of relevant structural models to gain quantitative information concerning the effect of concentration and temperature on intermolecular hydrogen bonding.

## 1. Introduction

Special attention has been paid to hydrogen bonding in alcoholic systems for two main reasons. The first reason is their potential use as model systems for the study of the more perplexed structures of strongly associated liquids. The second reason is that alcohols are used for the study of additional hydrogen-bonding-related effects, which are also exhibited in complex biological systems [1,2,3,4]. Furthermore, alcohols demonstrate amphiphilic characteristics and thus can be used to clarify the hydrophilic–hydrophobic interactions and their associations with macroscopic properties of the liquid state.

FTIR spectroscopy may provide important information on these issues due to its ability to identify structures formed via hydrogen bonding. Higher complexes are formed at higher concentrations and this fact is reflected in the concentration-dependent vibrational spectra of the solutions. Representative examples of the complexation mechanisms are the dimerization and polymerization that occur in alcoholic solutions, which are manifested in the OH stretching region of the infrared spectrum through substantial spectral changes [5,6,7,8,9,10,11].

Even though both water and alcohol are hydrogen-bonded systems, they demonstrate several substantial differences [12,13,14]. The structure of water is a three-dimensional network; on the other hand, alcohols have one possible donated hydrogen bond per molecule and two possible accepted hydrogen bonds, exhibiting a structure with reduced dimensionality compared with the structure of water. Furthermore, alcohol molecules are composed of a polar hydroxyl hydrophilic part and a non-polar alkyl hydrophobic part. The amphiphilic nature of alcohols further perplexes the hydrogen bond dynamics of these systems. Modulation of the hydrophobicity can be realized by varying the hydrophobic part of the intermolecular hydrogen bonding network. Hydrophobicity modulation can be accomplished by going from primary to tertiary alcohols and/or by changing the alkyl chain length. One last difference between water and alcohols is the miscibility of the latter with both non-polar and polar solvents. To advance our understanding of the self-association of alcohols, we chose CCl_4_ instead of water as a solvent. Isopentyl alcohol was chosen not only due to its biological significance [15] or catalytic dehydrogenation [16] but also due to the limited number of vibrational studies performed on this relatively simple primary alcohol.

In this study, we recorded the FTIR spectra of isopentyl alcohol dissolved in CCl_4_ as a function of concentration and temperature. The results were analyzed in view of the self-association model with the aim of quantitatively analyzing the effect of temperature and concentration on intermolecular hydrogen bonding. The experimental results are complemented with Density Functional Theory (DFT) calculations and second (MP2) and fourth (MP4) order Møller–Plesset perturbation theory calculations to elucidate the thermodynamic and vibrational properties of the species involved in the structure.

## 2. Results and Discussion

### 2.1. Short-Range Order: Concentration Effect

**Figure 1:**
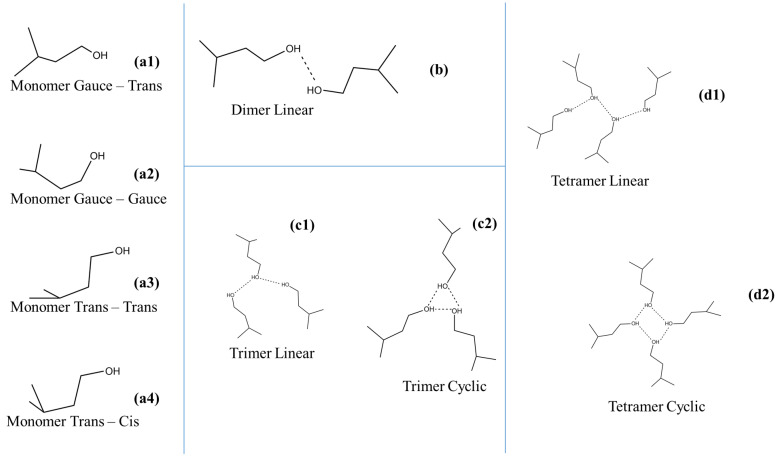
Schematic representation of the associated and unassociated species that are present in isopentyl alcohol–CCl_4_ solutions.

The overall structure of isopentyl alcohol–CCl_4_ can be considered as a typical self-associated alcoholic system composed of monomeric groups and associated monomers forming linear dimers and higher order oligomers, such as trimers and tetramers in linear and cyclic configuration. Higher-order oligomers in the structure are hard to stabilize. These species participate in equilibrium expressions, which are strongly affected by solution concentration and temperature variations. Furthermore, monomeric units exhibit distinct rotational trans–trans, gauche–gauche, gauche–trans and trans–cis isomers due to the rotation of the OH bond around the C–O axis. A schematic representation of these species is presented in Figure 1. If associations are occurring in the system, drastic spectral changes are detected in the corresponding spectra, revealing the extent of hydrogen bonding in the system. The concentration range studied in this work allowed us to detect the spectral fingerprint of all the above-reported species. Furthermore, it is important to note that the spectra mirror the association bonds in the system but not the individual associated species in the solution.

An unassociated OH functional group is present at one end of each oligomer species, independently of the number of monomeric units required to form the polymeric species. This means that the corresponding band will have contributions from all similar oligomers with unassociated OH groups. On the other hand, the associated OH group is hydrogen-bonded at the hydrogen site on the other end of the associated species. The associated OH group may also be found in both oxygen and hydrogen sites in the interior of associated species composed of three or more monomeric units. Thus, two distinct bands correlated with OH-associated groups are expected in the spectra due to the OH stretching mode. Another interesting feature is that the bandwidth of the associated species is always wider than that of the unassociated species.

**Figure 2:**
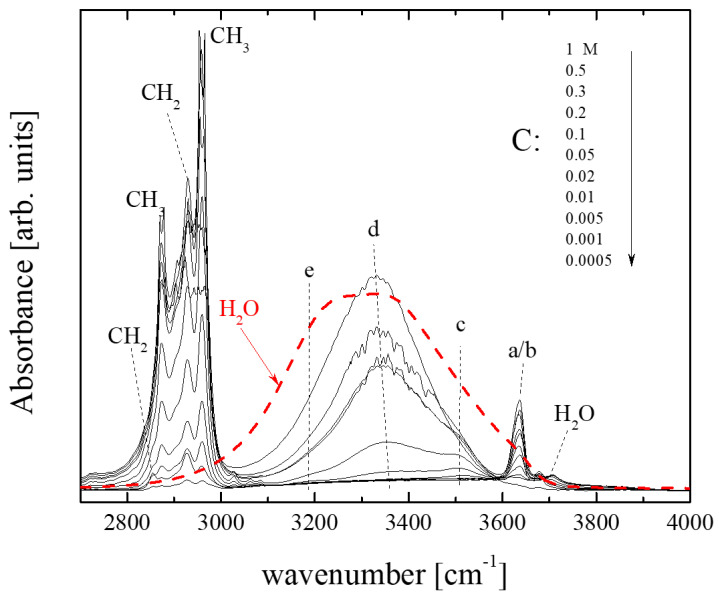
Infrared absorption spectra in the stretching frequency region of –CH_2_, –CH_3_ and –OH functional groups of all concentrations studied at 20 °C. The spectrum of pure H_2_O is also shown for comparison (dashed line). The letters a, b, c, d and e correspond to the individual peaks that fit the experimental spectra and are assigned to specific species. See the main text for a detailed assignment.

Figure 2 illustrates the infrared absorption spectra of isopentyl alcohol–CCl_4_ solutions in the high-frequency region of interest for all concentrations studied at room temperature. The spectrum of pure H_2_O is also shown for comparison. The sharp intense bands in the 2800–3000 cm^−1^ range correspond to CH_2_ and CH_3_ functional groups. At higher wavenumbers, a broad spectral envelope consisting of discrete contributions appears [17]. The broad bands at ~3200 and 3380 cm^−1^, denoted as e and d in Figure 2, are assigned to hydroxylic groups in the internal positions of hydrogen-bonded oligomers [17]. The band at ~3500 cm^−1^, denoted as c, is assigned to hydrogen-bond donor end groups of the open-chain oligomers. These bands are also present in the highly concentrated solutions, although with slightly red-shifted frequencies [17]. The asymmetry of the band at ~3600 cm^−1^ implies that two different spectral contributions can be resolved at this frequency range, namely the low-frequency band b appearing at ~3622 cm^−1^ and the high-frequency counterpart, band a, presented at ~3638 cm^−1^. The exact frequencies are strongly dependent on several factors, including the type of alcohol, the dilution level, the temperature, etc. The high-frequency counterpart (band a) is attributed to hydrogen-bond acceptor molecules at the end site of the open chain oligomers, while the low-frequency band (band b) is assigned to hydroxylic groups of non-hydrogen-bonded molecules [17]. The weak band at ~3800 cm^−1^ is attributed to H_2_O traces [18,19,20].

**Figure 3:**
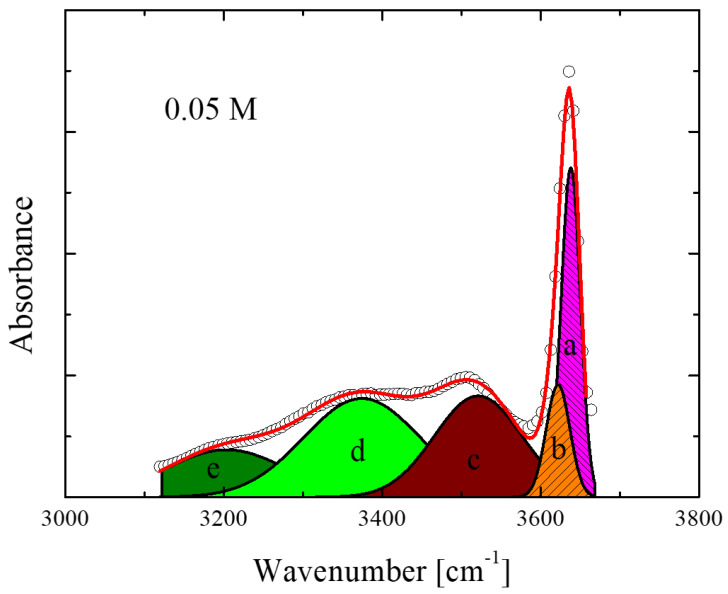
Representative fitting of the infrared absorption spectra in the OH stretching region. Open circles, thick solid lines and thin lines represent experimental data, total curve fit and individual vibrational peaks, respectively. The letters a, b, c, d and e correspond to the individual peaks that fit the experimental spectra and are assigned to specific species. See the main text for a detailed assignment.

A representative fitting example of the spectra for the 0.05 M solution is presented in Figure 3. The fitting procedure was performed using a sum of five Voigt profiles using the non-linear least squares Levenberg-Marquardt method. Five individual Voigt profiles are required to successfully fit the experimental spectra of all concentrations and temperatures studied and are in line with the structural model discussed above. Our assignments are supported by the outcome of the theoretical calculations described earlier.

In the context of the self-association model, two equilibrium constants are considered to describe the dimerization and polymerization reactions taking place in alcohols [21]. The mechanisms are formulated by Equations (1) and (2), respectively.

(1) Mo+Mo ↔Di

(2) $$\mathrm{Mo}+(\mathrm{Mo}{)}_{n}\;↔{\left(\mathrm{Mo}\right)}_{n+1}$$

The n and n+1 subscripts correspond to complexes of the n-order and (n+1)-order, respectively. The two equilibrium constants accounting for the dimerization and polymerization reactions [21] are:

(3) $$K_{1}=\frac{\left[Di\right]}{{\left[Mo\right]}^{2}}$$

and

(4) $$K_{c}=\frac{\left[{\left(Mo\right)}_{n+1}\right]}{\left[Mo\right]\left[{\left(Mo\right)}_{n}\right]}\times \frac{n}{n+1}$$

In the context of this model, all dissociation constants are not equal to each other, while a distinct constant is used for the dissociation of the dimer (*K*_1_) and a general dissociation constant (*K_c_*) is considered for the rest of the dissociation steps. The polymer units may be considered as cyclic species and/or linear chains.

**Figure 4:**
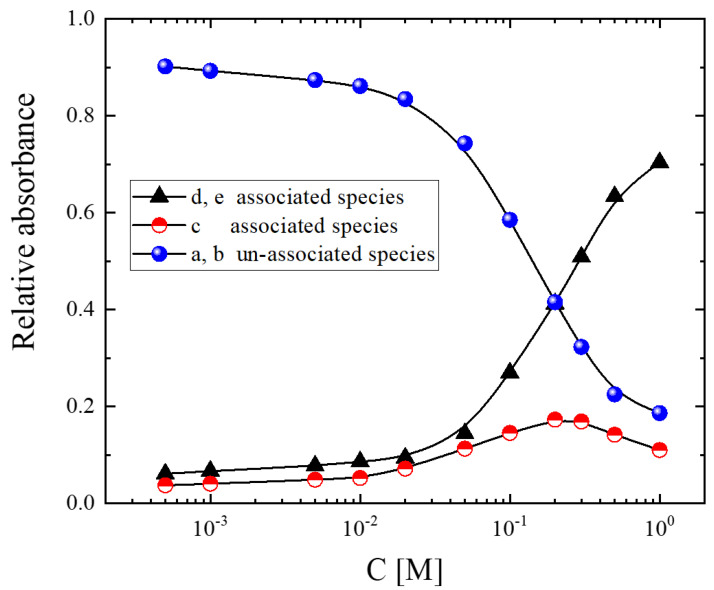
Relative absorbance of associated and unassociated species that are present in the structure of isopentyl alcohol–CCl_4_ solutions as a function of concentration. The calculation is based on the fitting procedure described in the main text. Lines are drawn as guides for the eye.

In the context of the above association model, we can estimate from the spectroscopic data the relative amounts of the different associated and unassociated species participating in the structure as a function of the alcohol concentration at room temperature. The results of all the concentrations studied are shown in Figure 4. In highly diluted solutions, the system consists almost entirely of unassociated species. With increasing concentration, the fraction of unassociated species decreases drastically, with a parallel increase in the associated species. More specifically, an increase in the stoichiometric alcohol concentration causes the absorbance of the c band assigned to associated units passing through a maximum. In the highly concentrated alcoholic solutions, the d and e bands corresponding to the associated species dominate the spectra.

**Figure 5:**
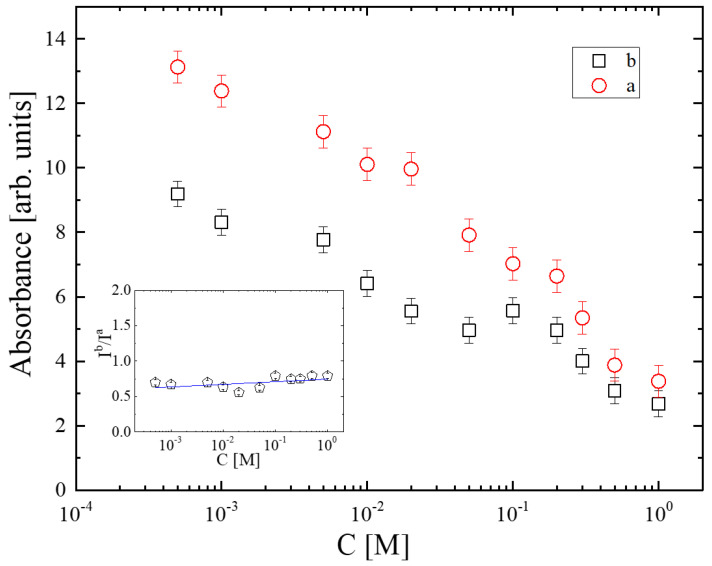
Concentration dependence of the a and b sub-bands as a function of solution concentration. Inset: variation in the I^b^/I^a^ absorbance ratio with concentration.

The relative absorbances of the a and b sub-bands as functions of solution concentration are presented in Figure 5. The concentration dependence reveals that both bands decrease monotonically. Furthermore, the inset of Figure 5 shows the variation in I^b^/I^a^ absorbance ratio with concentration. The ratio remains almost constant with increasing concentration, indicating that the decreasing trends of the a and b bands are similar. The reduction in both a and b spectral profiles correlated with the unassociated species implies a substantial degree of self-aggregation in the system.

**Figure 6:**
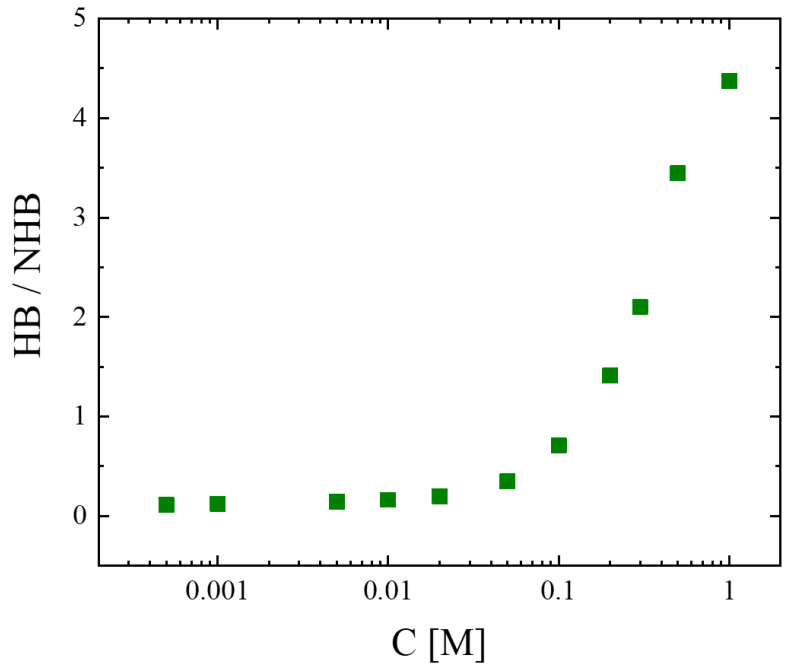
Fraction of band absorbances correlated with associated species and unassociated species as a function of concentration. Green symbols represent the experimental values.

To evaluate the extent of hydrogen bonding, and thus the self-association degree in the system, we estimated the fraction of the absorbances of bands attributed to the associated species to the absorbances of the bands attributed to the unassociated species; the results are illustrated in Figure 6. It seems that diluting isopentyl alcohol with the nonpolar solvent CCl_4_ reduces the number of hydrogen bonds, and thus the self-association degree, in the system. Hydrogen bonding becomes significant for concentrations above 0.1 M.

### 2.2. Short-Range Order: Temperature Effect

**Figure 7:**
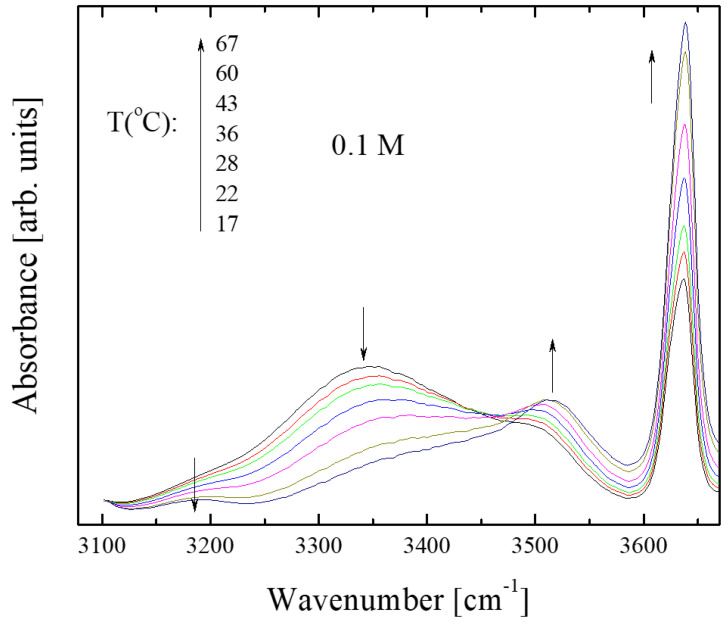
Infrared absorption spectra in the frequency region of interest as a function of temperature for 0.1 M solution of isopentyl alcohol dissolved in carbon tetrachloride. Arrows denote the trend of the peak absorbance with increasing temperature.

Another intriguing point was to evaluate the effect of temperature on hydrogen bonding in isopentyl alcohol solutions. We recorded the IR spectra of 0.1 M and 0.0005 M solutions of isopentyl alcohol dissolved in CCl_4_ over a range of temperatures, from 17 to 67 °C. Representative spectra for the 0.1 M solution are shown in Figure 7; the corresponding spectra for the 0.0005 M solution are completely isomorphous.

Figure 7 shows that increasing temperature affects the relative population of the associated and unassociated species in a manner analogous to dissolution. More specifically, it seems that an increase in temperature increases the population of unassociated species at the expense of associated units. To quantitatively follow these structural alterations as a function of temperature, we estimated the absorbance variation of the sub-bands discussed earlier.

**Figure 8:**
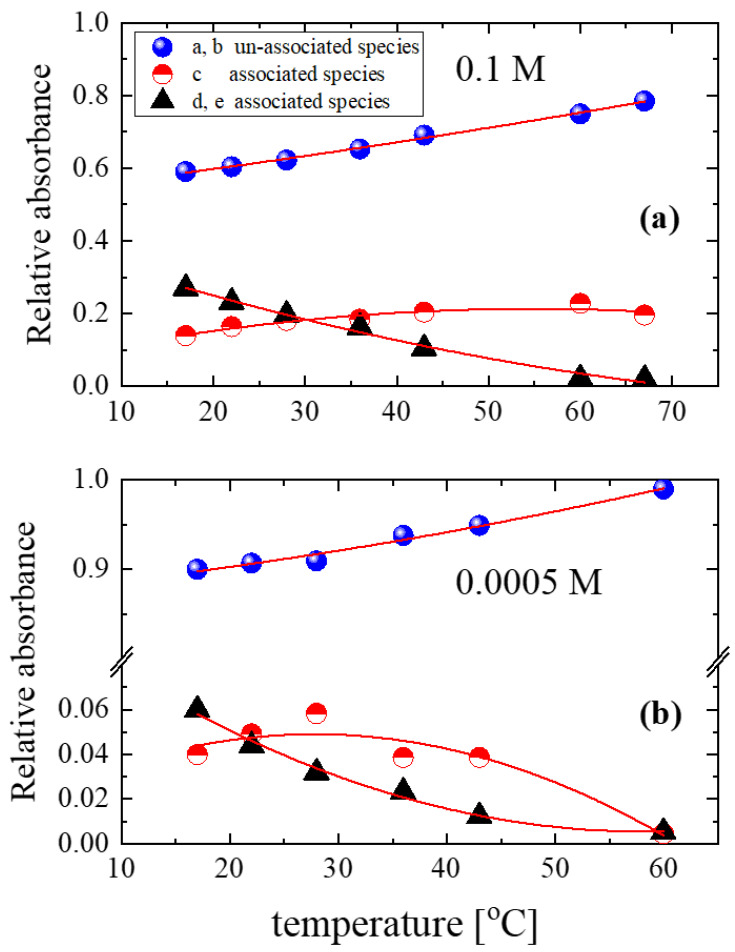
Relative absorbances of the associated and unassociated species present in the structure of isopentyl alcohol–CCl_4_ solutions as a function of temperature. The concentrations of the solutions were 0.1 M (**a**) and 0.0005 M (**b**). The lines in the figure correspond to 2nd-order polynomial fittings and are used to show the trend in the experimental data.

The results for concentrations 0.1 M and 0.0005 M are shown in Figure 8a and Figure 8b, respectively. The population of the unassociated species was high even at the starting temperature (17 °C). The fraction of unassociated species increased with increasing temperature, while the population of the associated species decreased monotonically. Additionally, the absorbance of band c reached a maximum with increasing temperature; the maximum exhibited by this band appeared at different temperatures depending on the alcohol concentration.

To follow the variations in the non-hydrogen-bonded hydroxyls with respect to the hydrogen-bonded hydroxyls, we estimated the HB/NHB ratios for the 0.1 and 0.0005 M concentrations as a function of temperature to evaluate the average strength of the interactions in the alcoholic solutions.

**Figure 9:**
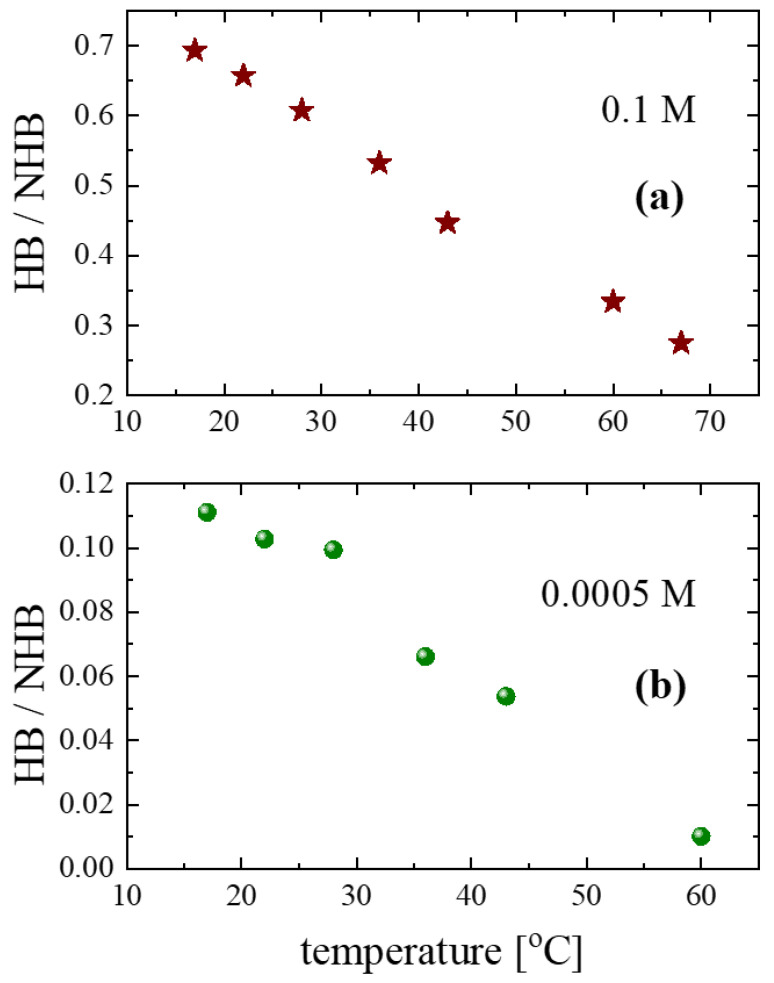
Temperature dependence of the HB/NHB ratio.

The results for 0.1 M and 0.0005 M are shown in Figure 9a and Figure 9b, respectively. For both concentrations, the ratio decreases monotonically as temperature increases. Furthermore, the value of the ratio for the 0.0005 M solution is lower than that of the 0.1 M solution, indicating the de-structuring effect that is induced by dissolving isopentyl alcohol in carbon tetrachloride and heating.

### 2.3. Theoretical Results

**Figure 10:**
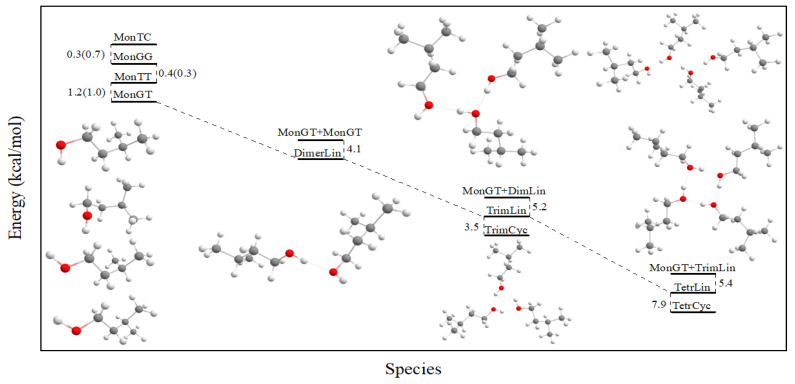
Isopentyl alcohol species and their energy differences in kcal/mol. Results in parentheses correspond to the MP2 method.

The structures of the monomer, dimer, trimer and tetramer conformers of isoamyl alcohol at 25 °C are displayed in Figure S1 of the Supplementary Materials. The conformers are denoted as gauche–trans, gauche–gauche, trans–trans and trans–cis. The first component of these words indicates the position of the C–O bond relative to the neighboring H_2_C–C bond, while the second component indicates the position of the O–H bond relative to the H_2_C–C bond. All structural parameters after optimization and the corresponding harmonic vibrational frequencies are presented in the Supplementary Materials and agree well with the reported results [22]. The differences in enthalpies at 25 °C are listed in Table 1 and displayed schematically in Figure 10 for B3LYP, MP2 and MP4 methods showing the stability order of the conformer structures. The gauche–trans conformer was found to be the most stable configuration.

As said, alcohols, like all molecules that easily form hydrogen bonds, are often represented by an infinite equilibrium model that considers associated species such as dimers, trimers and even tetramers [13,14,15,16,17,18,19,20,21,22,23,24,25,26]. Thus, to establish a correlation with our experimental findings, the association with isoamyl alcohol was considered. Three chain-like and two cyclic forms of isoamyl alcohol clusters were determined, i.e., a linear dimer, a linear and a cyclic trimer and a linear and a cyclic tetramer. The most stable conformer, i.e., the gauche–trans structure, was taken as the basic monomeric unit. The energy diagram of optimized structures of the associated species is presented in Figure 10.

The following association steps are assumed to occur in alcoholic solutions:

A_1_ + A_1_ ↔ A_2lin_ (5a)

A_2lin_ + A_1_ ↔ A_3lin_, A_3lin_ ↔ A_3cyc_ (5b)

A_3lin_ + A_1_ ↔ A_4lin_, A_4lin_ ↔ A_4cyc_ (5c)

where A_1_ represents the monomeric alcoholic unit. Accordingly, the stepwise stabilization enthalpies, ΔH, for building up the clusters at 298 K were calculated based on the following definitions:

ΔH _i,lin_ = H_i,lin_ − (H_i−1,lin_ + H_A1_) (6a)

ΔH _i,cyc_ = H_i,cyc_ − H_i lin_ (6b)

where the enthalpy values are calculated from the electronic energy results, with the inclusion of thermal enthalpy corrections at 298 K.

The results are depicted in Figure 10. Various conclusions can be drawn about the tendencies of the associations of the alcoholic molecules, which are directly correlated with distinct cooperative effects. Thus, the association energy of the dimerization is smaller than the enthalpy of adding a monomer to the dimer to form the linear trimer—5.2 kcal/mol for the latter versus 4.1 kcal/mol for the former. The step from the linear trimer to the linear tetramer provides a further slight increase in the formation enthalpy, though it is smaller than for the previous step. Consequently, further steps are not expected to provide any significant stabilization vs. the tetramers and are considered to have negligible probability of formation [27]. Another interesting finding concerns the stability of the cyclic clusters. The stabilities determined from the ab initio results indicate that cyclic clusters exist and present higher stabilization enthalpies than the values of the chain-like structures due to the higher number of hydrogen bonds. While cyclic dimers are not stable, cyclic trimers and cyclic tetramers (in particular) show significant stabilization, thus we can safely assume the existence of a certain percentage of the molecules in alcoholic solutions, which must be considered for the proper interpretation of the experimental measurements.

**Figure 11:**
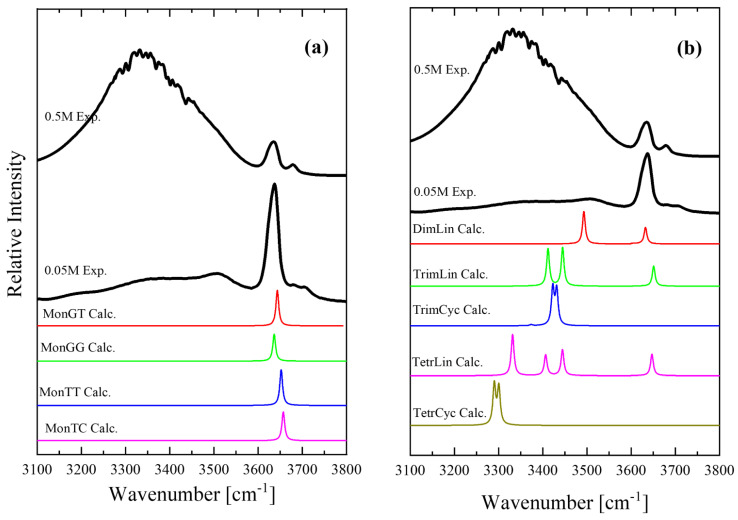
Comparison of the experimental and theoretical IR spectra of isopentyl alcohol monomer conformers (**a**) and oligomers and polymers, linear and cyclic species (**b**).

Figure 11 presents a direct comparison between the experimental and theoretical IR spectra of the isopentyl alcohol monomer conformers (Figure 11a) and the oligomers and polymers, linear and cyclic species (Figure 11b). Figure 11 also shows two different experimental spectra corresponding to concentrations of 0.05 and 0.5 M, with one in the dilute and one in the denser region. The experimental spectrum corresponding to the 0.05 M solution showed a close resemblance to the theoretical spectra of the monomer conformers. The theoretical spectra of the oligomers and polymers, linear and cyclic species (Figure 11b) support the methodology used for fitting the experimental spectra presented in Figure 3. There are also plenty of Molecular Dynamics (MD) simulations on this alcoholic system and even more complex systems. Molecular dynamics calculations have been carried out for aqueous solutions of isopropyl alcohol and its fluorinated compounds [28]. The promotion of water structure and the increase in hydrogen bonds between water molecules occurs not only near the fluoroalkyl group but also near hydroxyl groups in fluoroalcohols. The alcohol–water interaction is stronger for fluoroalcohols than for aliphatic alcohols owing to the electronegativity and the electron-withdrawing effect of fluorine atoms. The impact of surface hydrophilization on the adsorption of isopropyl alcohol solution in water has also been studied using MD [29]. In another MD study, the authors incorporated hydrogen bonding interactions between adsorbent and adsorbate (Metal-organic frameworks–isopropyl alcohol) and between adsorbate and adsorbate (isopropyl alcohol–isopropyl alcohol) to understand their impact on the transport of isopropyl alcohol molecules through pristine UiO-66 [30]. Molecular dynamics (MD) simulations were also performed on water and various alcohol liquids on a flat SiO_2_ surface terminated and by hydroxyl groups to examine the microscopic structures of these liquids near the solid surface and diffusion property for a fundamental understanding of the wet process during semiconductor fabrication [31].

## 3. Materials and Methods

### 3.1. Solutions

Anhydrous isopentyl alcohol (purity 99.7%) and anhydrous carbon tetrachloride (purity 99.8%) were purchased from Merck and Aldrich, respectively, and were used as received. Isopentyl alcohol–CCl_4_ solutions corresponding to concentrations of 1, 0.5, 0.3, 0.2, 0.1, 0.05, 0.02, 0.01, 0.005, 0.001 and 0.0005 M were prepared gravimetrically in alcohol. We chose to prepare the solutions in the high-dilution range (<1 M) to avoid potential interactions with the solvent (CCl_4_).

### 3.2. Spectroscopic Measurements

Fourier-transform infrared (FTIR) transmittance spectra were recorded in the 600–4000 cm^−1^ frequency region using a spectrometer (Bruker, Alpha model) with a resolution of 1 cm^−1^. Sixteen scans were averaged to obtain spectra with adequate signal-to-noise ratios. Temperature-dependent measurements were taken using a homemade high-temperature optical cell equipped with ZnSe windows and variable thicknesses adjusted using Teflon spacers in the range of 0.1–2 mm. Thickness depended only on the alcohol concentration to avoid the effect of total absorbance. Infrared absorption spectra were measured in the 17–67 °C temperature range, with an accuracy of ±0.5 °C, to avoid boiling the solutions. All measurements were taken immediately after the preparation of the samples to avoid evaporation. A detailed description of the experimental protocols followed to acquire high-temperature spectroscopic measurements in the liquid state has been reported elsewhere [32,33].

### 3.3. Theoretical Calculations

In addition to the experimental investigation, a computational characterization of the conformers of the isopentyl alcohol was carried out using ab initio and DFT methodologies. More specifically, the equilibrium structures, harmonic vibration frequencies and thermochemical data of the possible conformer structures were optimized at both the MP2 and B3LYP levels of theory and combined with the 6-311++G(d,p) basis set. The calculated structural, spectroscopic and energetic results from both methods were shown to be in good agreement with each other (see Supplementary Materials).

The self-association of the isoamyl alcohol molecules was also computationally investigated. Dimers, trimers and tetramers were calculated, and structural parameters and stabilization trends were examined for both linear and cyclic geometries. Because of the computational cost, the complete characterization of all clusters identified was carried out at the B3LYP/6-311++G(d,p) level of theory. However, since the present systems involve the investigation of molecular interactions of relatively weakly bonded complexes, the significance of the effect of the BSSE correction on the stability trends should be examined. MP2 and MP4 sp energy calculations at the B3LYP computed geometries, corrected with the inclusion of the BSSE correction according to the counterpoint method, were carried out [34]. The calculation was performed to estimate the magnitude of the effect in the stabilization energies of the linear and cyclic trimers. It turned out that the inclusion of the BSSE correction did not appreciably change the stabilization energy differences calculated at the B3LYP/6-311++G(d,p) level of theory without the inclusion of the BSSE correction due to mutual compensation cancellation. The effect can also be readily appreciated in the examination of the differences in the analytical stabilization energies of a series of alkanol–cyclohexane mixtures shown in Table 1 of the study by Vasiltsova and Heintz [27]. Thus, the overall stabilization trends of the determined clusters were based on the B3LYP/6-311++G(d,p) results.

All theoretical investigations were carried out using the Gaussian 09 algorithm [35].

## 4. Conclusions

In this work, we performed a detailed concentration- and temperature-dependent Fourier-transform infrared (FTIR) study of isopentyl alcohol dissolved in nonpolar carbon tetrachloride solvent. The infrared absorption measurements were performed within a temperature range below the boiling point of the solution. Emphasis was placed on the high-frequency -OH stretching region. The spectral features corresponding to associated and unassociated units, after decomposition of the spectra, were used to quantitatively follow the effect of temperature and concentration on intermolecular hydrogen bonding (HB) in the isopentyl alcohol. The experimental results were complemented with Density Functional Theory (DFT) calculations and second (MP2) and fourth (MP4) order Møller–Plesset perturbation theory calculations to elucidate the energetics and vibrational properties of the monomeric species in the structure. For the oligomeric and polymeric species, only the Density Functional Theory (DFT) was implemented due to the computational cost. The systematic examination of the theoretical and experimental results led us to the following conclusions: The overall structure of isopentyl alcohol–CCl_4_ solutions is a typical self-associated alcoholic system. The structure is dominated by the presence of monomeric groups and associated monomers that form linear dimers and higher-order oligomers such as trimers and tetramers in linear and cyclic configurations. Oligomers of higher order in the structure are hard to stabilize. These species participate in equilibrium expressions that are strongly influenced by solution concentration and temperature variations. Monomeric units show distinct rotational trans–trans, gauche–gauche, gauche–trans and trans–cis isomers due to the rotation of the OH bond around the C-O axis. The fraction of unassociated species increases with increasing concentration or/and temperature, while the population of the associated species decreases. The estimated HB/NHB ratio, indicative of the variation in the non-hydrogen-bonded hydroxyls with respect to the hydrogen-bonded hydroxyls, decreases with increasing temperature for both concentrations studied, indicating a de-structuring induced by the dissolution of isopentyl alcohol in carbon tetrachloride.

## Figures and Tables

**Table 1:** Angles (in degrees) and energies (in kcal/mol) of the isopentyl alcohol conformers. Percentages in parentheses indicate the relative populations of conformers in isopentyl alcohol. These values are from reference [22].

**Isopentyl Alcohol**				
	T_O_ (O–C–C–C)	T_C_ (C–C–C–H)	T_OH_ (H–O–C–C)	E_r_ (kcal/mol)
**Gauche–Trans (43%)**				
B3LYP	180.0	60.04	180.0	0
MP2	170.0	59.98	172.14	524.185
MP4	171.2	59.38	172.84	481.056
**Gauche–Gauche (32%)**				
B3LYP	65.57	171.81	61.14	1.750
MP2	61.61	166.86	59.53	525.659
MP4	62.22	167.85	59.58	482.594
**Trans–Trans (24%)**				
B3LYP	180.0	180.0	180.0	1.186
MP2	180.0	180.0	180.0	525.107
MP4	180.0	180.0	180.0	481.979
**Trans–Cis (1%)**				
B3LYP	180.0	180.0	0.02	2.221
MP2	180.0	180.0	0.05	526.456
MP4	177.79	178.83	56.45	482.173

## Data Availability

Data are available upon request from the corresponding author.

## Supplementary Materials

The following supporting information can be downloaded at: https://www.mdpi.com/article/10.3390/molecules28176285/s1, Table S1: Theoretically estimated bond angles (in degrees) of isopentyl alcohol species, Table S2: Theoretically estimated bond lengths (in Å) of isopentyl alcohol species, Table S3: Theoretically estimated vibrational frequencies, absorbances and assignments of isopentyl alcohol species, Figure S1. All isopentyl alcohol species studied theoretically with B3LYP and MP2 methods combined with the 6-311++G(d,p) basis set under tight optimization convergence criteria. Figure S2. Numerical labels for every atom referenced in tables for all the species

## Sample Availability

Samples of the compounds are available from the corresponding author.
