# Supplementary material for: Intermolecular Hydrogen Bonding in Associated Fluids: The Case of Isopentyl Alcohol Dissolved in Carbon Tetrachloride
Source: Molecules. 2023 Aug 28;28(17):6285. doi: 10.3390/molecules28176285 (PMC10488694; doi:10.3390/molecules28176285)
Supplement: Supplementary file 1 [file molecules-28-06285-s001.zip › molecules-2564614-supplementary.pdf]

Supplementary Material

Table S1. Theoretically estimated bond angles in degrees of isopentyl alcohol species.

Bond Angles					
Monomer	Dimer	Trimer Linear	Trimer Cyclic	Tetramer Linear	Tetramer Cyclic
$C_1 – O_1 – H_1$ 108.84370	$C_1 – O_1 – H_1$ 109.16476	$C_1 – O_1 – H_1$ 108.79752	$C_1 – O_1 – H_1$ 109.98788	$C_1 – O_1 – H_1$ 108.91035	$C_1 – O_2 – H_1$ 109.60459
	$O_2 – H_1 – O_1$ 173.60617	$O_1 – H_1 – O_2$ 173.32284	$O_1 – H_1 – O_2$ 151.12786	$O_2 – H_1 – O_2$ 175.87228	$O_1 – H_1 – O_2$ 168.44131
	$O_1 – H_1 – O_2$ 114.05106	$H_1 – O_2 – C_2$ 108.53733	$H_1 – O_2 – H_2$ 89.30643	$H_1 – O_2 – C_2$ 118.00322	$O_2 – H_1 – O_2$ 126.67114
	$H_1 – O_2 – H_2$ 115.12623	$H_1 – O_2 – C_2$ 125.49091	$H_1 – O_2 – C_2$ 129.28839	$H_1 – O_2 – H_2$ 107.60269	$H_1 – O_2 – H_1$ 103.27911
	$C_1 – O_2 – H_2$ 108.97792	$C_2 – O_2 – H_2$ 109.29531	$C_2 – O_2 – H_2$ 109.84308	$C_2 – O_2 – H_2$ 109.07306	$C_2 – O_2 – H_1$ 109.83239
		$O_2 – H_2 – O_3$ 176.05082	$O_2 – H_2 – O_3$ 150.82392	$O_2 – H_2 – O_3$ 177.51606	$O_2 – H_2 – O_3$ 166.34535
		$H_2 – O_3 – H_3$ 117.23291	$H_2 – O_3 – H_3$ 90.14539	$H_2 – O_3 – H_3$ 118.00495	$H_2 – O_3 – C_3$ 128.69265
		$H_2 – O_3 – C_3$ 121.05136	$H_2 – O_3 – C_3$ 133.33619	$H_2 – O_3 – C_3$ 122.42563	$H_2 – O_3 – H_3$ 101.44097
		$H_3 – O_3 – C_3$ 109.00036	$C_3 – O_3 – H_3$ 110.14370	$C_3 – O_3 – H_3$ 109.68170	$C_3 – O_3 – H_3$ 109.48655
			$O_2 – H_3 – O_1$ 149.08706	$O_2 – H_3 – O_4$ 176.94970	$O_3 – H_3 – O_4$ 168.79464
			$H_3 – O_1 – C_1$ 134.54156	$H_3 – O_4 – H_4$ 114.57735	$H_3 – O_4 – C_4$ 126.30049
			$H_3 – O_2 – H_1$ 89.20555	$H_3 – O_4 – C_4$ 119.96025	$H_3 – O_4 – H_4$ 103.04650
				$C_4 – O_4 – H_4$ 109.13215	$C_4 – O_4 – H_4$ 109.61308

					$O_4 - H_4 - O_1$ 166.70078
					$H_4 - O_1 - C_1$ 128.65398
					$H_4 - O_1 - H_1$ 101.63722

Table S2. Theoretically estimated bond lengths in Å of isopentyl alcohol species.

Bond Lengths					
Monomer	Dimer	Trimer Linear	Trimer Cyclic	Tetramer Linear	Tetrameric Cyclic
$C_1 - O_1$ 1.43199	$C_2 - O_2$ 1.43831	$C_1 - O_1$ 1.42330	$C_1 - O_1$ 1.42969	$C_1 - O_1$ 1.42231	$C_1 - O_1$ 1.42969
$O_1 - H_1$ 0.96169	$O_2 - H_2$ 0.96335	$O_1 - H_1$ 0.97423	$O_1 - H_1$ 0.97423	$O_1 - H_1$ 0.97619	$O_1 - H_1$ 0.98242
	$O_2 \cdots H_1$ 1.90886	$O_2 \cdots H_1$ 1.83884	$O_2 \cdots H_1$ 1.88963	$O_2 \cdots H_1$ 1.84921	$O_2 \cdots H_1$ 1.77638
	$C_1 - O_1$ 1.42405	$C_2 - O_2$ 1.42986	$C_2 - O_2$ 1.43129	$C_2 - O_2$ 1.42816	$C_2 - O_2$ 1.42946
	$O_1 - H_1$ 0.97055	$O_2 - H_2$ 0.97454	$O_2 - H_2$ 0.97581	$O_2 - H_2$ 0.97934	$O_2 - H_2$ 0.98209
		$C_3 - O_3$ 1.44625	$O_3 \cdots H_2$ 1.89083	$O_3 \cdots H_2$ 1.80379	$O_4 \cdots H_2$ 1.79623
		$O_3 - H_3$ 0.96219	$C_3 - O_3$ 1.42984	$C_3 - O_3$ 1.43694	$C_4 - O_4$ 1.43694
		$O_3 \cdots H_2$ 1.86937	$O_3 - H_3$ 0.97543	$O_3 - H_3$ 0.97351	$O_4 - H_4$ 0.98225
			$O_1 \cdots H_3$ 1.91345	$O_4 \cdots H_3$ 1.85935	$O_4 \cdots H_3$ 1.77451
				$C_4 - O_4$ 1.44442	$C_3 - O_3$ 1.42986
				$O_4 - H_4$ 0.96260	$O_3 - H_3$ 0.98268
					$O_1 \cdots H_4$ 1.79278

Table S3. Theoretically estimated vibrational frequencies, absorbances and assignments of isopentyl alcohol species.

Monomer	wavenumber	3843.18 cm^{-1}			
	intensities	29.2230			
	assignment	Stretching Free – OH			
Dimer	wavenumber	3676.80 cm^{-1}	3823.49 cm^{-1}		
	intensities	629.1217	31.3851		
	assignment	Stretching HB $O-H\cdots O$	Stretching Free-OH		
Trimer Linear	wavenumber	3591.35 cm^{-1}	3626.13 cm^{-1}		
	intensities	724.8467	746.9326		
	assignment	Stretching HB Hydrogens	Stretching HB Hydrogens		
Trimer Cyclic	wavenumber	3551.20 cm^{-1}	3602.66 cm^{-1}	3612.03 cm^{-1}	
	intensities	30.1374	1075.3198	1019.0011	
	assignment	Stretching HB Hydrogens	Stretching HB Hydrogens	Stretching HB Hydrogens	
Tetramer Linear	wavenumber	3506.92 cm^{-1}	3585.68 cm^{-1}	3625.74 cm^{-1}	3838.92 cm^{-1}
	intensities	1194.1500	602.5587	750.4350	41.5125
	assignment	Stretching HB Hydrogens	Stretching HB Hydrogens	Stretching HB Hydrogens	Stretching Free – OH
Tetramer Cyclic	wavenumber	3392.43 cm^{-1}	3463.34 cm^{-1}	3474.19 cm^{-1}	3501.07 cm^{-1}
	intensities	2.8271	2346.7304	2182.4804	5.2990
	assignment	Stretching HB Hydrogens	Stretching HB Hydrogens	Stretching HB Hydrogens	Stretching HB Hydrogens

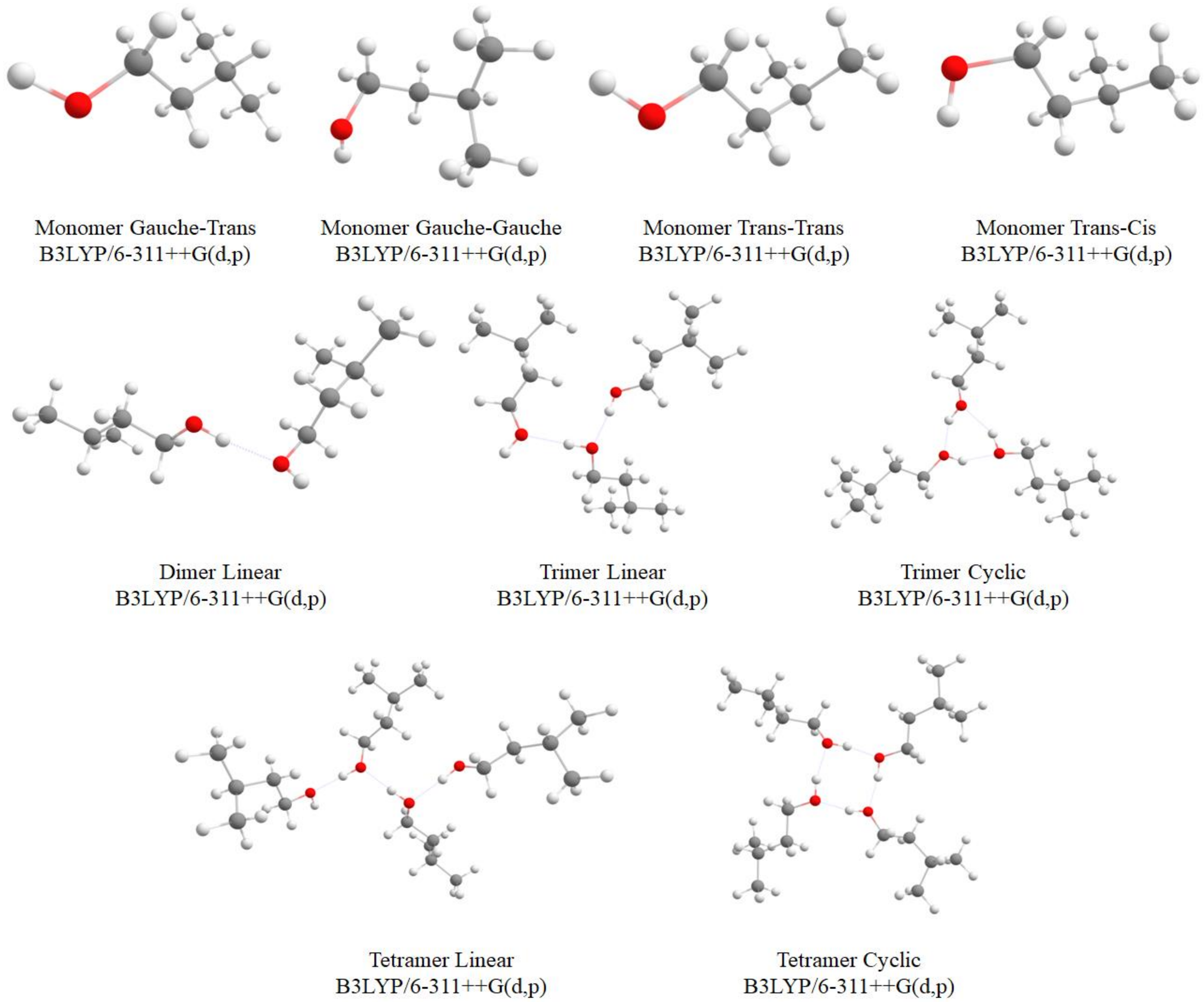

Figure S1. All isopentyl alcohol species studied theoretically with B3LYP and MP2 methods combined with the 6-311++G(d,p) basis set under tight optimization convergence criteria.

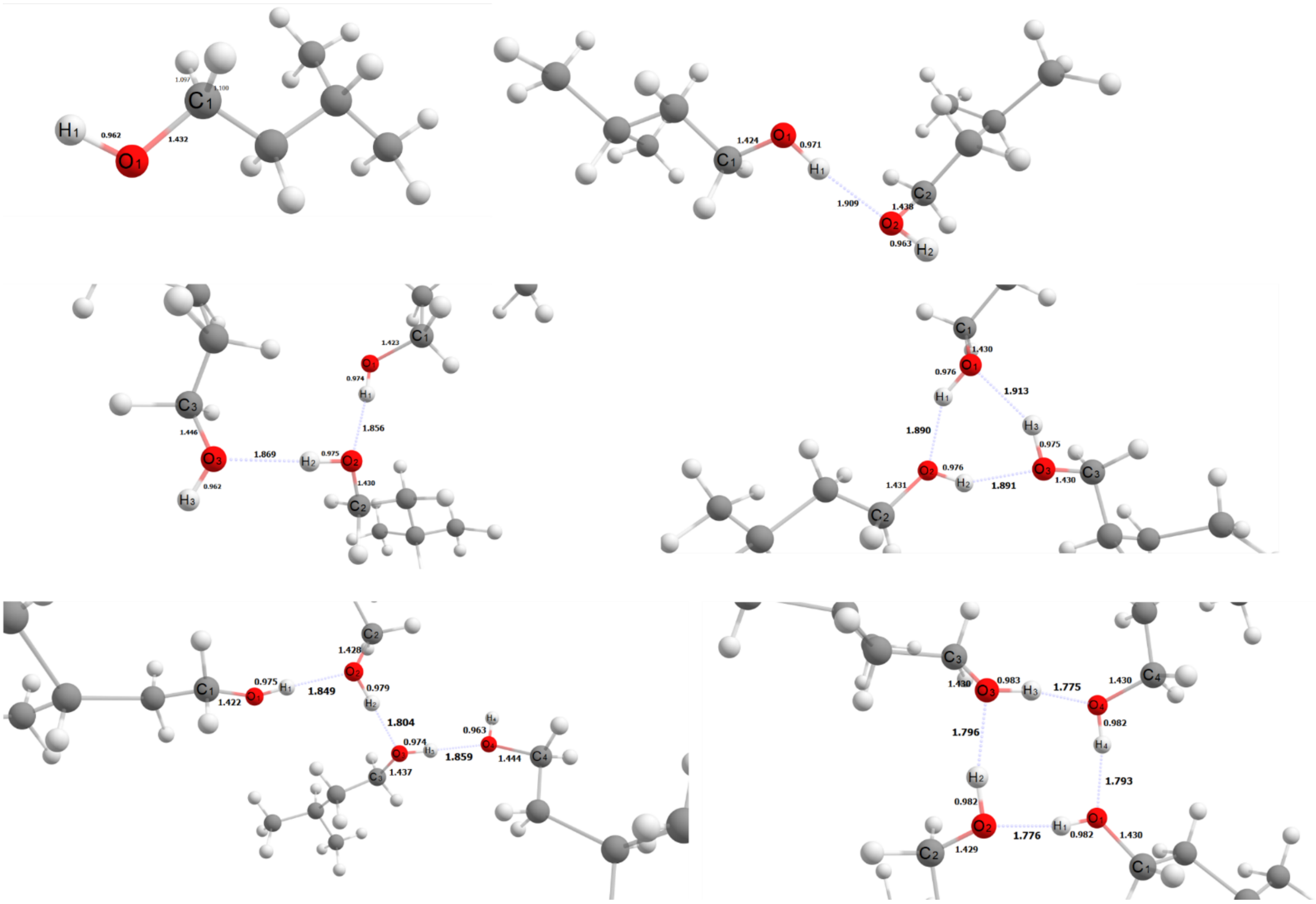

Figure S2. Numerical labels for every atom referenced in tables for all the species
